# On the relationship between the heritability and the attributable fraction

**DOI:** 10.1007/s00439-019-02006-8

**Published:** 2019-04-02

**Authors:** Elisabeth Dahlqwist, Patrik K. E. Magnusson, Yudi Pawitan, Arvid Sjölander

**Affiliations:** Karolinska Institute, Nobels väg 12A, 171 77 Stockholm, Sweden

## Abstract

Heritability is the most commonly used measure of genetic contribution to disease outcomes. Being the fraction of the variance of latent trait liability attributable to genetic factors, heritability of binary traits is a difficult technical concept that is sometimes misinterpreted as the more-easily understandable concept of attributable fraction. In this paper we use the liability threshold model to describe the analytical relationship between heritability and attributable fraction. Towards this end, we consider a hypothetical intervention that is aimed to reduce the genetic risk of the disease for a specified target group of the population. We show how the relation between the heritability and the attributable fraction depends on the disease prevalence, the intervention effect and the size of the target group. We use two real examples to illustrate the practical implications of our theoretical results.

## Introduction

Measuring the genetic influence on human diseases is one of the most important topics in medicine and epidemiology. This is commonly done in terms of the heritability, routinely reported using studies of siblings and twins (Lichtenstein et al. 2000; Mucci et al. 2016). For continuous traits, heritability is defined as the proportion of phenotypic variation that can be attributed to genetic variation. This definition can also be used for binary traits; however, for binary traits it is more common to define the heritability as the proportion of variance on the latent liability scale attributed to genetic variation, which is conceptually more difficult and often misinterpreted (Visscher et al. 2008; Witte et al. 2014). For example, in attempts to assess how much of a disease burden can be attributed to genetic factors, heritability is sometimes misinterpreted as an attributable fraction (AF) (Mucci et al. 2016). The AF for a particular exposure is defined as the proportion of disease cases that would be prevented if the exposure was eliminated from the population (Levin 1953). This definition differs from the heritability in two important aspects. First, in contrast to the AF, the heritability does not measure the effect of an intervention. Second, while the AF refers to a specific and well-defined exposure, which (in principle) can be eliminated, the heritability captures, in a loose sense, the aggregated impact of variation over the whole genome on the disease.

Despite differences between the concepts, it is intuitively reasonable that the heritability conveys something meaningful about the impact of genetic interventions. At one extreme, when the heritability is equal to 1, we would expect genetic interventions to have a large potential for reducing the disease prevalence. At the other extreme, when it is zero, genetic interventions will not have any impact on the disease prevalence. However, no formal analysis of the relationship between the overall heritability and the AF exists. Previous studies have either restricted the interest to the heritability attributed to a limited set of SNPs (Wang et al. 2018) or adressed the overall heritability but lacked a general formalization of this relationship (Ramakrishnan and Thacker 2012). In this work, we derive a formal link between the overall heritability and the AF by using the liability threshold model (Falconer 1965) and an extension of the AF which allows for continuous exposures (Morgenstern and Bursic 1982; Taguri et al. 2012).

The outline of this paper is as follows. First, we review the theory behind the liability threshold model and use the model to derive the relation between the AF and the heritability. Next, we illustrate the practical implications of this relationship with two real examples: one concerns the prevention of cardiovascular events by medication, and the other a comparison between two strategies for breast cancer prevention.

## Theory

### The liability threshold model

Falconer (1965) developed the liability threshold model to explain how a large number of environmental and genetic factors result in a dichotomous disease outbreak. The model assumes that the binary observed disease status *Y* can be described by a continuous latent (i.e. unobserved) liability *L*, which captures all genetic and environmental risk factors that influence the disease risk. Let *G* and *E* be scalar measures that summarize these genetic and environmental risk factors, respectively; we hereafter refer to *G* and *E* as the genetic and environmental ‘risks’. The liability model assumes that

1 $$\begin{aligned} L=G+E. \end{aligned}$$

Moreover, it assumes that the disease occurs when the subject’s liability *L* exceeds a threshold $\beta$, so that the disease prevalence $p(Y=1)$ is equal to $p(L> \beta )$.

The epidemiological literature distinguishes between the point prevalence (i.e. the proportion of the population that has the disease at a given point in time) and the lifetime prevalence (i.e. the proportion of the population that develops the disease at some point during life). Here, we are interested in ever experiencing the disease and we are thus interested in the lifetime prevalence.

Before proceeding, we emphasize an important feature of the liability threshold model. Because the model is additive, it is, for any given value $E<\infty$, possible to find values of *G* for which the liability falls below the threshold $\beta$. In particular, this happens when $G=-\infty$. The model thus assumes that there exists an ‘optimal’ genetic composition that will prevent the disease from occurring, regardless of what environment the subject is exposed to. For multifactorial diseases, this assumption may be reasonable as an approximation to reality, at least when the environment is not too extreme. We note, though, that even for multifactorial diseases one can easily conceive of extreme environments that would cause the disease, regardless of the subject’s genetic composition (e.g. living inside a nuclear reactor will always cause cancer).

In line with the literature (Falconer and Mackay 1996), we assume that *G* and *E* are independent and normally distributed with mean zero and variances $\sigma ^2_G$ and $\sigma ^2_E$, respectively. By assuming that *G* and *E* are linearly related to *L* as in (1), it follows that the liability is normally distributed with mean zero and variance $\sigma ^2_G+\sigma ^2_E$. Moreover, the joint distribution of the liability *L* and the genetic risk *G* is bivariate normal:

2 $$\begin{aligned} \left( \begin{array}{c}G\\ L\end{array}\right) \sim N\left\{ \left( \begin{array}{c}0\\ 0\end{array}\right) ,\left( \begin{array}{cc}\sigma _G^2 &{} \sigma ^2_G\\ \sigma ^2_G &{} \sigma ^2_G+\sigma ^2_E\end{array}\right) \right\} . \end{aligned}$$

### The heritability as a parameter in the liability threshold model

The heritability ($h^2$) is defined as the ratio of genetic variation and phenotypic variation, or variation in liability,

3 $$\begin{aligned} h^2=\frac{\sigma ^2_G}{\sigma ^2_G+\sigma ^2_E}. \end{aligned}$$

Hence, the correlation between the liability and the genetic risk is the square root of the heritability,

4 $$\begin{aligned} \text {Corr}(L,G)= & {} \frac{\text {Cov}(L,G)}{\sqrt{\text {Var}(L)\text {Var}(G)}}=\frac{\sigma _G^2}{\sigma _G\sqrt{\sigma _G^2+\sigma _E^2}}\nonumber \\= & {} \frac{\sigma _G}{\sqrt{\sigma _G^2+\sigma _E^2}}=h. \end{aligned}$$

Thus, the joint distribution of the genetic risk and the liability is a function of the heritability. In the next section, we show how this can be used to link the AF with the heritability.

### The attributable fraction

The AF is a commonly used measure in epidemiology for quantifying the impact of an exposure on an outcome (Levin 1953). The AF is defined as

5 $$\begin{aligned} \text {AF}=\frac{p-p^*}{p}, \end{aligned}$$

where *p* is the proportion of subjects that will ever get the disease in the factual situation, and $p^*$ is the counterfactual proportion that will ever get the disease under an intervention that eliminates the exposure from the population.

The AF measures the (net) proportion of disease cases prevented by the intervention. For instance, suppose that the factual prevalence is 5%, and that the intervention reduces the prevalence to 1%. The proportion of prevented disease cases is then equal to $(0.05-0.01)/0.05=80\%$.

In our context, the exposure of interest is the genetic risk *G* in the liability model. This is supposed to capture the aggregated impact of variation over the whole genome on the disease, which cannot be ‘eliminated’ in any meaningful sense. We can, however, think of hypothetical interventions that aim at manipulating the genetic risk in other ways, e.g. by changing its distribution or shifting it by a fixed constant. Thus, we use the standard definition of the AF in (5), but allow for $p^*$ to represent the counterfactual disease prevalence under such ‘generalized’ interventions. This generalization of the AF is sometimes referred to as the generalized impact fraction (Morgenstern and Bursic 1982; Taguri et al. 2012).

In practice, interventions may be targeted towards subgroups of the population due to, for instance, considerations of cost-effectiveness. For example, even though preventive medication against high blood pressure and cholesterol have been shown to reduce the risk of cardiovascular events (Yusuf et al. 2016), it might not be possible to implement this intervention on the whole population, since those at low risk will not have a sufficiently high benefit to motivate bearing the cost of the medication. Instead, it is more efficient to target the intervention to those at the highest genetic risk of a cardiovascular event (Tada et al. 2016).

We allow for targeted interventions that only apply to subjects with particularly high genetic risks, e.g. those who have a genetic risk above a certain quantile in the genetic risk distribution. We thus define the target group as those subjects for whom $G>b\sigma _G$, where *b* is a fixed constant that corresponds to a quantile in the standard normal distribution. For instance, $b=1.64$ and $b=1.96$ correspond to 0.95 and 0.975 quantiles, respectively, and setting *b* to $-\infty$ implies that we include the whole population in the target group. In practice, a high genetic risk group could be identified by using familial risk or a genetic risk score (Yoon et al. 2002; Belsky et al. 2013; Khera et al. 2016; Tada et al. 2016).

We consider interventions that reduce the genetic risk *G* with an amount $k\sigma _G$, where *k* is a fixed positive constant. Defining $L^*$ as the liability under the intervention we thus have that

$$\begin{aligned} L^*= G - k\sigma _G + E. \end{aligned}$$

If the intervention is targeted to a subgroup of the population, the counterfactual disease prevalence $p^*$ in (5) can be divided into two components. For subjects who belong to the target group, the genetic risk is reduced to $G-k\sigma _G$, whereas for subjects outside the target group the genetic risk is unchanged. The counterfactual (joint) probability of developing the disease and being in the target group is thus $p(L^*> \beta , G> b\sigma _G)=p(L> \beta + k\sigma _G, G > b\sigma _G)$, where

6 $$\begin{aligned}&p(L> \beta + k\sigma _G, G> b\sigma _G) \nonumber \\&\quad = p\left( \frac{L}{\sqrt{\sigma _G^2+\sigma _E^2}}> \frac{\beta }{\sqrt{\sigma _G^2+\sigma _E^2}}+\frac{k\sigma _G}{\sqrt{\sigma _G^2+\sigma _E^2}}, \frac{G}{\sigma _G}> b\right) \nonumber \\&\quad = p\left( \frac{L}{\sqrt{\sigma _G^2+\sigma _E^2}}> \frac{\beta }{\sqrt{\sigma _G^2+\sigma _E^2}} + kh, \frac{G}{\sigma _G} > b\right) . \end{aligned}$$

Since the intervention is only given to subjects with genetic risk *G* above the threshold $b\sigma _G$, the difference between the factual (observed) prevalence *p* and counterfactual (under the intervention) prevalence *p* arises only because of shifted liability levels within this target group. Thus, without loss of generalization we can express the numerator in (5) as

7 $$\begin{aligned} \begin{aligned} p-p^*&= p(L> \beta ,G> b\sigma _G)-p(L-k\sigma _G>\beta ,G> b\sigma _G)\\&= p\left( \frac{L}{\sqrt{\sigma _G^2+\sigma _E^2}}> \frac{\beta }{\sqrt{\sigma _G^2+\sigma _E^2}},\frac{G}{\sigma _G}> b\right) \\&\quad -p\left( \frac{L}{\sqrt{\sigma _G^2+\sigma _E^2}}> \frac{\beta }{\sqrt{\sigma _G^2+\sigma _E^2}} + kh, \frac{G}{\sigma _G} > b\right) . \end{aligned} \end{aligned}$$

Furthermore, we have that

8 $$\begin{aligned} p &= p(L>\beta )\nonumber \\&= p\left( \frac{L}{\sqrt{\sigma _G^2+\sigma _E^2}}>\frac{\beta }{\sqrt{\sigma _G^2+\sigma _E^2}}\right) \nonumber \\&= \varPhi \left( -\frac{\beta }{\sqrt{\sigma _G^2+\sigma _E^2}}\right) , \end{aligned}$$

where $\varPhi (\cdot )$ denotes the standard (i.e. mean 0, variance 1) normal distribution function. The bivariate standard normal distribution function with correlation coefficient *h* is denoted as $\varPhi (\cdot ,\cdot ;h)$. We can then express the difference in prevalence as

9 $$\begin{aligned} p-p^* = \varPhi \{\varPhi ^{-1}(p),-b;h\}-\varPhi \{\varPhi ^{-1}(p)-kh,-b;h\}. \end{aligned}$$

The expression in (9) shows how the disease prevalence is modified by the intervention, *k*, the size of the target group, *b*, and the square root of the heritability, *h*. We refer to Appendix A for a detailed derivation of (9).

From (9), the AF can be written as

10 $$\begin{aligned} \text {AF}(p, b, k, h) = \frac{\varPhi \{\varPhi ^{-1}(p),-b;h\}-\varPhi \{\varPhi ^{-1}(p)-kh,-b;h\}}{p}. \end{aligned}$$

In practice, we may set the constant *k* depending on what reduction in disease prevalence we imagine a given intervention would result in for a fixed heritability and size of the target group. For example, suppose that the intervention is given to the whole population ($b=-\infty$), so that the expression in (9) simplifies to $\varPhi \{\varPhi ^{-1}(p)\}-\varPhi \{\varPhi ^{-1}(p)-kh\}$. Suppose further that the disease prevalence is 5% before the intervention and 2.5% after the intervention. The reduction $\varDelta$ is then the difference between the 0.95 and 0.975 quantiles of the standard normal distribution, i.e. $\varDelta =1.96-1.64=0.32$. For a particular heritability, say $h^2=0.64$, this implies that $k=\frac{0.32}{0.8}=0.4$. Thus, a 2.5 percentage point reduction in disease prevalence for a disease with a heritability of 64% corresponds to $k=0.4$. Moreover, setting *k* to $\infty$ reduces *G* to $-\infty$, thus optimizing the genetic composition so that the disease is guaranteed to be prevented.

### Properties of the attributable fraction

The expression in (10) depends on four parameters: the intervention effect, *k*, the size of the target group, *b*, the heritability $h^2$, and the disease prevalence, *p*. When *k* increases, the second term in the numerator increases as well. Thus, the AF increases monotonically with *k*, which is intuitively reasonable. At the one extreme $k=0$ (no intervention), the two terms in the numerator become equal, so that the AF becomes equal to 0. At the other extreme $k=\infty$ (the genetic composition is optimized for those in the target group) the second term in the numerator equals 0, so that the AF simplifies to

11 $$\begin{aligned} \text {AF}(p, b, k=\infty , h^2) =\frac{\varPhi \{\varPhi ^{-1}(p),-b;h\}}{p}. \end{aligned}$$

It is not immediately obvious from the expression in (10) how the AF depends on *b*, but it can be shown (see Appendix B) that the AF decreases monotonically with *b*. This is intuitively reasonable; the smaller the target group, the smaller is the impact of the intervention. At the one extreme $b=\infty$ (no one is targeted by the intervention), both terms in the numerator of (10) equal 0, so that the AF equals 0 as well. At the other extreme $b=-\infty$ (the whole population is included in the target group), both terms in the numerator of (10) simplify to univariate distribution functions, so that the AF simplifies to

12 $$\begin{aligned} \text {AF}(p, b= & {} -\infty , k, h^2)\nonumber \\= & {} \frac{\varPhi \{\varPhi ^{-1}(p)\}-\varPhi \{\varPhi ^{-1}(p)-kh\}}{p}\nonumber \\= & {} \frac{p-\varPhi \{\varPhi ^{-1}(p)-kh\}}{p}. \end{aligned}$$

When both $k=\infty$ and $b=-\infty$, it can be seen from either (11) or (12) that the AF equals 1. This makes intuitive sense; if the genetic composition is optimized for everybody in the population, then 100% of all disease cases would be prevented.

**Fig. 1:**
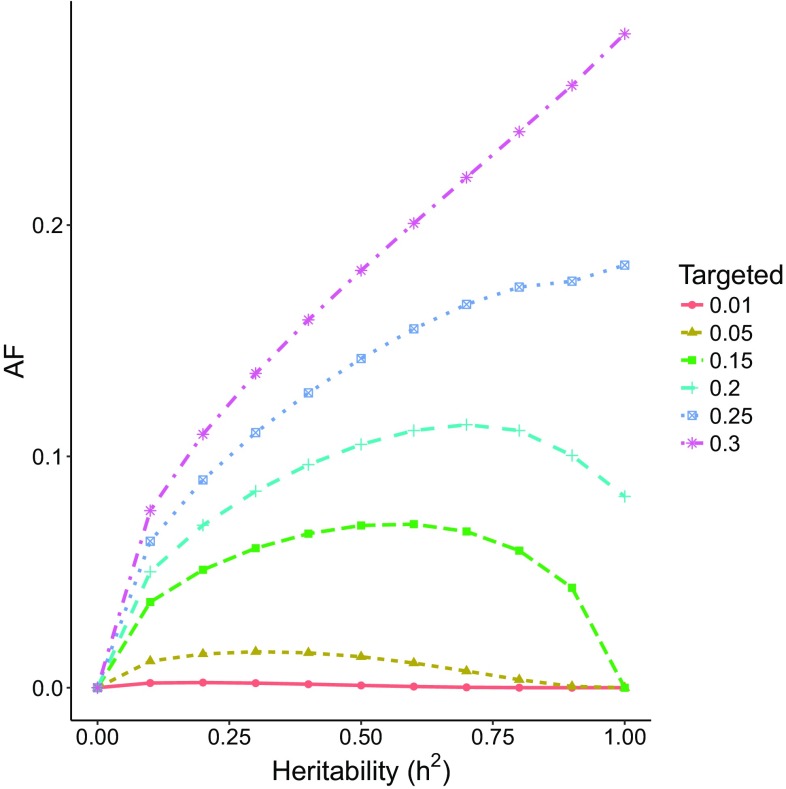
The AF as a function of $h^2$ for $k=1,p=0.5$ and target group of between 1 and 30% of those at the highest genetic risk

One would perhaps expect that the AF increases monotonically with $h^2$; i.e. the more heritable the disease, the larger is the impact of genetic interventions. However, the AF is not (necessarily) a monotone function of $h^2$. Figure 1 shows the AF as a function of $h^2$ for $k=1, p=0.5$ and with the target group of the intervention ranging between 1 and 30% of those at the highest genetic risk. For small target groups, the AF increases up to $h^2$ between 0.6 and 0.8, and then starts to decrease. However, we see that for large target groups (25% or larger), the relationship between AF and $h^2$ is monotone. In general, if the intervention is given to the whole population and $b=-\infty$, we observe from the expression in (12) that the AF does indeed increase monotonically with $h^2$.

We note that the hypothetical scenario in Fig. 1 is unrealistic, in that the disease prevalence is unusually high.

The relationship between the AF and the disease prevalence is rather intricate, as the prevalence appears in both numerator and denominator of the expression in (10). When $p=1$ (everybody gets the disease), both terms in the numerator of (10) simplify to the univariate distribution function $\varPhi (-b)$, so that the AF equals 0. When *p* approaches 0, it can be shown that the AF approaches 1 (see Appendix B). We have made an extensive grid search over *k*, *b*, $h^2$ and *p*. Based on this grid search, we conjecture that the AF decreases monotonically with *p*; however, we have not been able to prove this analytically.

## Examples

In the previous section, we have derived the theoretical relationship between the heritability and the AF, by means of a hypothetical intervention that reduces the genetic risk of a disease. In this section, we illustrate the implications of this relationship through practical examples. In particular, we show how we can use the relationship to investigate the population impact of various intervention strategies.

### Example 1: blood pressure and cholesterol-lowering medication

High blood pressure and cholesterol levels are well-known risk factors for cardiovascular events such as acute myocardial infarction (AMI) and stroke (Mozaffarian et al. 2015; Khera et al. 2016; Yusuf et al. 2016). These risk factors both have strong genetic components (Weissglas-Volkov and Pajukanta 2010; van Rijn et al. 2007) and can be lowered by preventive medical treatment with statins (cholesterol lowering) and angiotensin II receptor antagonists (blood pressure lowering) (Yusuf et al. 2016).

In a Swedish study, the heritability of AMI was estimated to be 36% and the prevalence of AMI in this cohort was approximately 6% (Zdravkovic et al. 2007). In a Danish study, the heritability for stroke was estimated to be 17% and the estimated prevalence of stroke in this cohort was around 4% (Bak et al. 2002).

We will now use these examples to investigate how the differences in heritability and prevalence between AMI and stroke impact the AF. We compare how the population impact differs between the diseases depending on if the intervention is given to those at 1% or 5% highest genetic risk of AMI and stroke, i.e. $b=2.33$ or $b=1.65,$ respectively. The target groups could possibly be identified by a genetic risk score developed for cardiovascular disease (Thanassoulis et al. 2012).

If the intervention is given to 5% at the highest genetic risk, we suppose that this intervention can reduce the prevalence by 1.1 percentage points (i.e. from 6% to 4.9%) for AMI and 0.4 percentage points (i.e. from 4% to 3.6%) for stroke. Such a genetic risk reduction corresponds to an intervention effect of $k=1$ for both diseases.

**Fig. 2:**
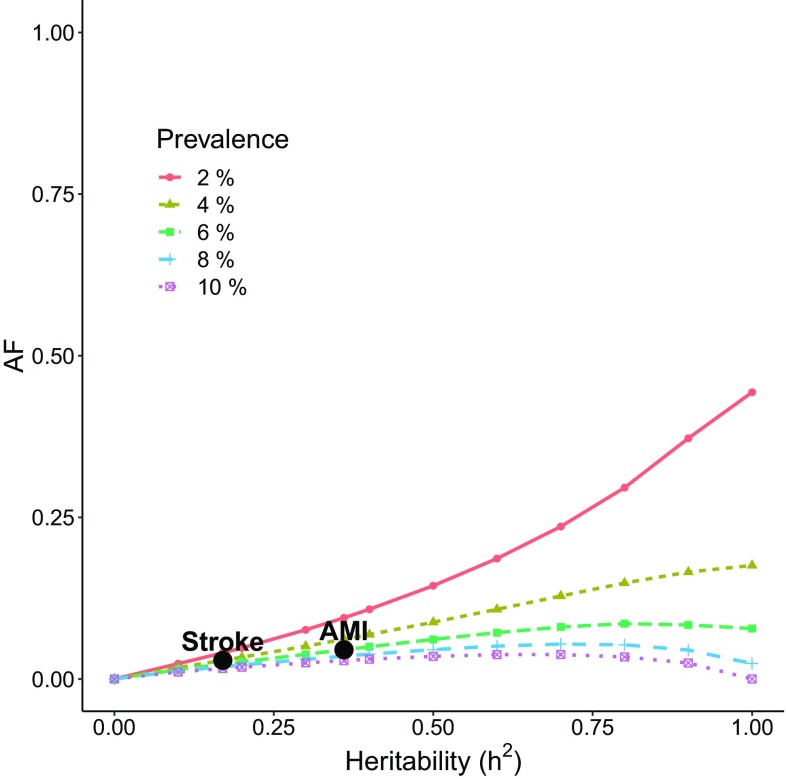
The AF as a function of heritability for intervention given to the 1% at the highest genetic risk. The intervention effect is assumed to be $k=1$

**Fig. 3:**
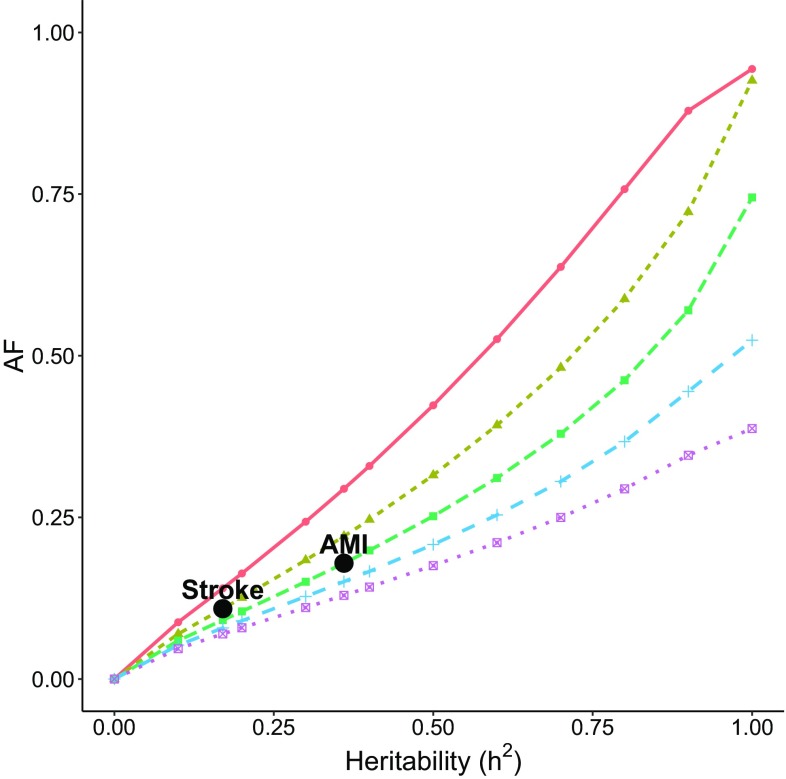
The AF as a function of heritability for intervention given to the 5% at the highest genetic risk. The intervention effect is assumed to be $k=1$

Figures 2 and 3 illustrate the AF as a function of heritability and prevalence. The prevalences are 2, 4, 6, 8 and 10% and the intervention effect is fixed at $k=1$. In Fig. 2, the intervention is targeted at the 1% at highest genetic risk and in Fig. 3 it is given to the 5% highest genetic risk.

The examples of AMI and stroke are marked in Figs. 2 and 3. If the intervention is given to 1% of the population, the AF is 4.5% for AMI and 2.9% for stroke. When the intervention is given to 5% of the population, the AF is 17.9% for AMI and 10.9% for stroke. From these two examples we observe how an intervention with larger coverage may drastically increase the population impact of the intervention. We note, however, that the benefit of increasing the target group eventually levels off, as the intervention also covers subjects with small genetic risk who do not benefit from the intervention. In general, we also observe that the smaller the prevalence, the larger is the AF for a fixed heritability, target group and intervention effect. These, and other features of the relationships between the heritability, intervention effect, target group size and disease prevalence can be investigated using our Shiny app ‘afheritability’ (Dahlqwist et al. 2018).

### Example 2: prevention of breast cancer

Breast cancer is one of the most common cancers in women, with a lifetime prevalence of 8.1% and a heritability of 31%, estimated from Swedish twin data (Möller et al. 2016). Prevention strategies for breast cancer include screening programs and specialized treatments for those women at the highest genetic risk of breast cancer (Moyer and U.S. Preventive Services Task Force 2014). For example, women who are carriers of the BRCA1/2 mutations are often offered surgical removal of all breast tissue susceptible to cancer, bilateral prophylactic mastectomy (BPM) (Rebbeck et al. 2004). Another prevention strategy is treatment with tamoxifen (Moyer and US Preventive Services Task Force 2013, 2014). However, none of these interventions are used on a large scale since tamoxifen have severe side effects and may increase the risk of other adverse health outcomes (Nichols et al. 2015), and BPM is an invasive surgical procedure that may lead to additional complications (Moyer and US Preventive Services Task Force 2013). In this example we are interested in the difference in population impact of these two interventions for a fixed heritability and prevalence of breast cancer.

**Fig. 4:**
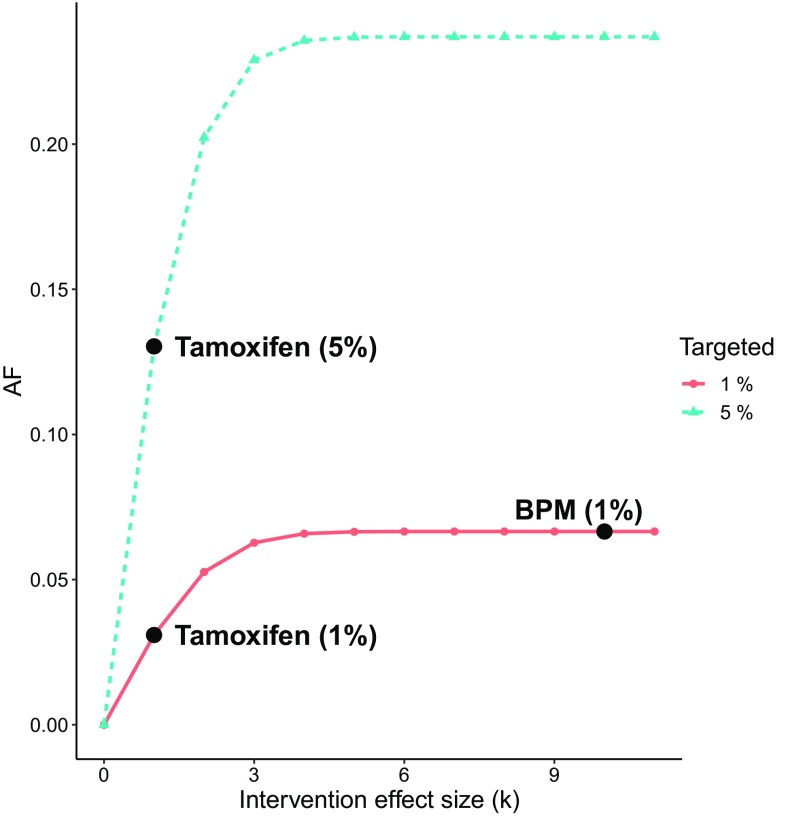
The AF for breast cancer with $h^2=31\%$ and $p=8.1\%$. Comparison of BPM given to a target group of 1% with $k=10$ and tamoxifen given to target group of 1% versus 5% with $k=1$

Studies have shown that BPM almost eliminates the risk of breast cancer (Rebbeck et al. 2004). We thus assume that the intervention effect of the BPM intervention almost eliminates breast cancer within the target group, e.g. $k=10$. Moreover, preventive treatment with tamoxifen has been shown to almost halve the cumulative rate of invasive breast cancer (Fisher et al. 2005). Thus, based on these studies we assume that preventive tamoxifen treatment can reduce the lifetime prevalence within the target group by around 50%, which approximately corresponds to $k=1$.

The target groups for these interventions can be chosen based on a genetic risk score for breast cancer (Shieh et al. 2016). We assume that BPM will only be given to women at the highest 1% genetic risk of breast cancer, i.e. $b=2.33$. Tamoxifen treatment is not as invasive as the BPM intervention. However, due to the adverse effects of tamoxifen (Fisher et al. 1994; van Leeuwen et al. 1994), it is only recommended to women with a high genetic risk of breast cancer (Moyer and US Preventive Services Task Force 2013). Therefore we assume that that this intervention can be given to, at most, women at the highest 5% genetic risk of breast cancer, i.e. $b=1.64$. We compare the AF for tamoxifen given to those at the 1% versus 5% highest genetic risk.

Figure 4 illustrates the AF as a function of the intervention effect *k*, for heritability 31% and target group sizes equal to 1% and 5%. We observe that the AF is 6.7% for the BPM intervention given to the 1% at the highest genetic risk, 3.1% for the tamoxifen intervention given to the 1% at the highest genetic risk and 13% for the tamoxifen intervention given to the 5% at the highest genetic risk. Thus, even though BPM almost eliminates breast cancer within the target group, it has a smaller impact than the less efficient tamoxifen intervention given to 5%, but larger impact than tamoxife intervention given to 1%. This example illustrates that a large effect of a prevention strategy may not have a large population impact if the intervention is limited to a small part of those at risk.

## Discussion

Heritability is a central concept in genetic epidemiology. Yet, because it is defined in terms of proportion of variance of latent disease liability, it is difficult to interpret the population implications of a particular value of the heritability (Witte et al. 2014). Attempts to do so sometimes interprets heritability as an attributable fraction (AF) (Mucci et al. 2016). In this article, we have shown how the relationship between the heritability and the AF can be formalized and how these results can be used to understand the population implications of a particular value of the heritability.

The relationship between the heritability and the AF is rather intricate, since it depends on several parameters in a non-linear way. Both the disease prevalence and the effect of the genetic intervention modify the impact of the heritability on the AF. In reality, interventions may not always be targeted to the whole population. We have accounted for this by adding the possibility to consider situations where the intervention is only targeted at those at the highest genetic risk. Intuitively, one would expect that the AF increases monotonically with the heritability. However, we have shown by examples that this is not necessarily the case. In particular, we have shown that, if the prevalence is high and the target group is small, then the AF may increase with the heritability up to a certain point, after which it will start to decrease.

Two examples have been used to illustrate how our results can be used to understand the relationship between the heritability and the AF for different scenarios. The first example is an intervention with blood pressure and cholesterol-lowering medication to prevent AMI and stroke. In this example we have considered the target group, *b*, and the intervention effect, *k*, to be fixed and we have compared the AF separately for AMI and stroke. For the same intervention, the AF is larger for AMI compared to stroke due to the larger heritability of AMI. In the second example, we have compared two interventions to prevent breast cancer, bilateral prophylactic mastectomy and tamoxifen with a fixed heritability, $h^2$, and prevalence, *p*. Bilateral prophylactic mastectomy (BPM) has a large intervention effect, but can only be given to a limited target group. Compared to BPM, tamoxifen has a smaller intervention effect but can be given to a larger target group. In this example, we observed how the AF is larger for the tamoxifen intervention compared to the BPM intervention despite its lower intervention effect since the target group is larger.

We are not the first to use the AF to measure the effect of genes. However, virtually all applications that we are aware of define the exposure as a single SNP/gene (Claus et al. 1996; Witte et al. 2014; Khoury et al. 2005) or a limited set of SNPs (Wang et al. 2018), thus measuring the effect of that single SNP and not the aggregated impact of the whole genome. A notable exception is Ramakrishnan and Thacker (2012), who used the AF for twin data and defined the exposure for a given twin as the disease status in the co-twin. The authors claimed that, by using this exposure definition, their attributable fraction would measure the proportion of disease cases that are ‘due to heritability’. However, they did not formally motivate this claim, and it is not obvious whether the term ‘due to heritability’ is empirically meaningful.

To derive the relation between the heritability and the AF, we have used the liability threshold model, which relies on several important assumptions. Specifically, it assumes that the genetic and environmental risks for the disease are normally distributed, the effect of genes and environment add up to the liability (i.e. that there is no additive statistical interaction between the risk factors) and the genetic and environmental risks are independent. Regarding the first assumption, it is reasonable to assume that the genetic and environmental risks are normally distributed, since we are considering complex traits that depend on the accumulated small contributions from many different genetic and environmental factors. The second assumption of mainly additive effects from the genetic and environmental factors has been debated to a great extent (Hill et al. 2008). However, there is not much evidence of statistical interaction effects between genes and environment (Hill et al. 2008; Hunter 2005). The third assumption of no gene–environment correlation is violated if genes affect the environment or vice versa. Gene–environment correlation might occur due to various reasons (Jaffee and Price 2008) and should be carefully considered in each particular application.

In this article, we have conceptualized ‘genetic interventions’ as all interventions that modify the genetic risk. These can be pure genetic interventions, such as gene therapy, or interventions that target the mechanisms by which the genes exert their effect, such as bilateral prophylactic mastectomy. Thus, by ‘genetic’ we do not necessarily mean that the genes are modified per se, but rather that the intervention modifies the circumstances that allow the genetic variants to manifest.

Throughout, we have defined the target group as those at the highest genetic risk. As suggested by one of the reviewers, target groups may in practice also be defined in terms of environmental risk for disease. In Appendix C we derive the relation between the AF and the heritability, when the target groups are defined as those subjects for which the total (genetic and environmental) liability exceeds a certain threshold. We show that, in fact, this alternative definition of the target group leads to simpler calculations, which only involves univariate normal distribution functions.

## Appendix A: Proof of (9)

We have that

$$\begin{aligned} p-p^* &= p(L>\beta )-p(L^*>\beta )\\ &= \{p(L>\beta ,G>b\sigma _G)+p(L>\beta ,G\le b\sigma _G)\}\\&-\{p(L^*>\beta ,G>b\sigma _G)+p(L^*>\beta ,G\le b\sigma _G)\}\\&= p(L>\beta ,G>b\sigma _G)-p(L^*>\beta ,G>b\sigma _G)\\&= p(L>\beta ,G>b\sigma _G)-p(L-k\sigma _G>\beta ,G>b\sigma _G)\\&= p\left( \frac{L}{\sqrt{\sigma _G^2+\sigma _E^2}}>\frac{\beta }{\sqrt{\sigma _G^2+\sigma _E^2}},\frac{G}{\sigma _G}>b\right) \\&-p\left( \frac{L}{\sqrt{\sigma _G^2+\sigma _E^2}}>\frac{\beta +k\sigma _G}{\sqrt{\sigma _G^2+\sigma _E^2}},\frac{G}{\sigma _G}>b\right) \\&= p\left( \frac{L}{\sqrt{\sigma _G^2+\sigma _E^2}}>-\varPhi ^{-1}(p),\frac{G}{\sigma _G}>b\right) \\&-p\left( \frac{L}{\sqrt{\sigma _G^2+\sigma _E^2}}>-\varPhi ^{-1}(p)+kh,\frac{G}{\sigma _G}>b\right) \\&= \varPhi \{\varPhi ^{-1}(p),-b;h\}-\varPhi \{\varPhi ^{-1}(p)-kh,-b;h\}, \end{aligned}$$

where the second last equality follows from (8), and the last equality follows from (2) and (4).

## Appendix B: Proof of relations between the AF and *k*, *b*, $h^2$ and *p*

Let *X* and *Y* be bivariate standard normal with correlation *h*. We then have the following:

- The AF is decreasing in *b*. *Proof* From (10) we have that
  $$\begin{aligned} \frac{\partial \text {AF}}{\partial b} &= -\frac{p\{X<\varPhi ^{-1}(p),Y=-b\}-p\{X<\varPhi ^{-1}(p)-kh,Y=-b\}}{p}\\& = -p(Y=-b)\frac{p\{X<\varPhi ^{-1}(p)|Y=-b\}-p\{X<\varPhi ^{-1}(p)-kh|Y=-b\}}{p}\\&= -\phi (b)\frac{\varPhi \left\{ \frac{\varPhi ^{-1}(p)+hb}{\sqrt{1-h^2}}\right\} -\varPhi \left\{ \frac{\varPhi ^{-1}(p)+h(b-k)}{\sqrt{1-h^2}}\right\} }{p}<0, \end{aligned}$$
  where the third equality follows from the conditional distribution of *X*, given *Y*, and *k* being positive.
- $\lim _{p\rightarrow 0}\text {AF}=1$. Proof: Define $q=\varPhi ^{-1}(p)$. We have that
  $$\begin{aligned} \lim _{p\rightarrow 0}\text {AF}= & {} \lim _{q\rightarrow -\infty }\text {AF}\\= & {} \lim _{q\rightarrow -\infty }\frac{\partial \{p(X<q,Y<-b)-p(X<q-kh,Y<-b)\}/\partial q}{\partial \varPhi (q)/\partial q}\\= & {} \lim _{q\rightarrow -\infty }\frac{p(X=q,Y<-b)-p(X=q-kh,Y<-b)}{\phi (q)}\\= & {} \lim _{q\rightarrow -\infty }\frac{p(X=q)p(Y<-b|X=q)-p(X=q-kh)p(Y<-b|X=q-kh)}{\phi (q)}\\= & {} \lim _{q\rightarrow -\infty }\frac{(2\pi )^{-1/2}\left[ \text {exp}(-q^2/2)\varPhi \left( \frac{-b-qh}{\sqrt{1-h^2}}\right) -\text {exp}\{-(q-kh)^2/2\}\varPhi \left\{ \frac{-b-(q-kh)h}{\sqrt{1-h^2}}\right\} \right] }{(2\pi )^{-1/2}\text {exp}(-q^2/2)}\\= & {} \lim _{q\rightarrow -\infty }\varPhi \left( \frac{-b-qh}{\sqrt{1-h^2}}\right) -\text {exp}[\{2qkh-(kh)^2\}/2]\varPhi \left\{ \frac{-b-(q-kh)h}{\sqrt{1-h^2}}\right\} \\= & {} 1 \end{aligned}$$
  where the second equality follows from L’Hospital’s rule, and the last equality follows by $\lim _{q\rightarrow -\infty }\varPhi \left( \frac{-b-qh}{\sqrt{1-h^2}}\right) =\lim _{q\rightarrow -\infty }\varPhi \left\{ \frac{-b-(q-kh)h}{\sqrt{1-h^2}}\right\} =1$ and $\lim _{q\rightarrow -\infty }\text {exp}[\{2qkh-(kh)^2\}/2]=0$.

## Appendix C: Relationship when the intervention is based on overall liability

To accommodate both genetic and environmental factors, we may target the intervention to subjects with $L>b\sqrt{\sigma _G^2+\sigma _E^2}$. We then have that

$$\begin{aligned} p-p^*&= p(L>\beta )-p(L^*>\beta )\\&= \left\{ p\left( L>\beta ,L>b\sqrt{\sigma _G^2+\sigma _E^2}\right) \right. \\&\quad \left. +p\left( L>\beta ,L\le b\sqrt{\sigma _G^2+\sigma _E^2}\right) \right\} \\&\phantom {11}-\left\{ p\left( L^*>\beta ,L>b\sqrt{\sigma _G^2+\sigma _E^2}\right) \right. \\&\quad \left. +p\left( L^*>\beta ,L\le b\sqrt{\sigma _G^2+\sigma _E^2}\right) \right\} \\& = p\left( L>\beta ,L>b\sqrt{\sigma _G^2+\sigma _E^2}\right) \\&\quad -p\left( L^*>\beta ,L>b\sqrt{\sigma _G^2+\sigma _E^2}\right) \\= & {} p\left( L>\beta ,L>b\sqrt{\sigma _G^2+\sigma _E^2}\right) \\&\quad -p\left( L-k\sigma _G>\beta ,L>b\sqrt{\sigma _G^2+\sigma _E^2}\right) \\= & {} p\left\{ L>\text {max}\left( \beta ,b\sqrt{\sigma _G^2+\sigma _E^2}\right) \right\} \\&\quad -p\left\{ L>\text {max}\left( \beta +k\sigma _G,b\sqrt{\sigma _G^2+\sigma _E^2}\right) \right\} \\= & {} p\left\{ \frac{L}{\sqrt{\sigma _G^2+\sigma _E^2}}> \text {max}\left( \frac{\beta }{\sqrt{\sigma _G^2+\sigma _E^2}},b\right) \right\} \\&\quad -p\left\{ \frac{L}{\sqrt{\sigma _G^2+\sigma _E^2}}>\text {max}\left( \frac{\beta +k\sigma _G}{\sqrt{\sigma _G^2+\sigma _E^2}},b\right) \right\} \\= & {} p\left[ \frac{L}{\sqrt{\sigma _G^2+\sigma _E^2}}>\text {max}\left\{ -\varPhi ^{-1}(p),b\right\} \right] \\&\quad -p\left[ \frac{L}{\sqrt{\sigma _G^2+\sigma _E^2}}>\text {max}\left\{ -\varPhi ^{-1}(p)+kh,b\right\} \right] \\= & {} \varPhi \left[ \text {min}\left\{ \varPhi ^{-1}(p),-b\right\} \right] \\&\quad -\varPhi \left[ \text {min}\left\{ \varPhi ^{-1}(p)-kh,-b\right\} \right] , \end{aligned}$$

where the second last equality follows from (3) and (8), and the last equality follows from (2). Thus, AF can be written as

$$\begin{aligned} \text {AF}(p,b,k,h)=\frac{\varPhi \left[ \text {min}\left\{ \varPhi ^{-1}(p),-b\right\} \right] -\varPhi \left[ \text {min}\left\{ \varPhi ^{-1}(p)-kh,-b\right\} \right] }{p}. \end{aligned}$$

We observe that, for this alternative definition of the target group, the AF becomes a relatively simple function of (*p*, *b*, *k*, *h*), since it only involves univariate normal distribution functions. Below, we investigate how the AF depends on (*p*, *b*, *k*, *h*).

We first note that

13 $$\begin{aligned} \varPhi ^{-1}(p)-kh>-b\Rightarrow \text {AF}=0, \end{aligned}$$

so in this case, AF does not depend on (*p*, *b*, *k*, *h*).

Dependency on *k*:

- $$\begin{aligned} k<\frac{\varPhi ^{-1}(p)+b}{h}\Rightarrow \text {AF}=0 \end{aligned}$$. Follows from (13).
- $$\begin{aligned} k>\frac{\varPhi ^{-1}(p)+b}{h}\end{aligned}$$: AF increases monotonically with *k*.
- $$\lim _{k\rightarrow \infty }=\frac{\varPhi \left[ \text {min}\left\{ \varPhi ^{-1}(p),-b\right\} \right] }{p}.$$

Dependency on *h*:

- $$\begin{aligned} h<\frac{\varPhi ^{-1}(p)+b}{k}\Rightarrow \text {AF}=0 \end{aligned}$$. Follows from (13).
- $h>\frac{\varPhi ^{-1}(p)+b}{k}$: AF increases monotonically with *h*.
- $$\begin{aligned} h=1\Rightarrow \text {AF}=\frac{\varPhi \left[ \text {min}\left\{ \varPhi ^{-1}(p),-b\right\} \right] -\varPhi \left[ \text {min}\left\{ \varPhi ^{-1}(p)-k,-b\right\} \right] }{p} \end{aligned}$$.

Dependency on *b*:

- $$\begin{aligned} b>-\varPhi ^{-1}(p)+kh\Rightarrow \text {AF}=0 \end{aligned}$$. Follows from (13).
- $-\varPhi ^{-1}(p)<b<-\varPhi ^{-1}(p)+kh\Rightarrow \text {AF}=\frac{\varPhi (-b)-\varPhi \left\{ \varPhi ^{-1}(p)-kh\right\} }{p},$ which decreases monotonically with *b*.
- $$\begin{aligned} b<-\varPhi ^{-1}(p)\Rightarrow \text {AF}=1-\frac{\varPhi \left\{ \varPhi ^{-1}(p)-kh\right\} }{p} \end{aligned}$$; doesn’t depend on *b*.

Dependency on *p*:

- $$\begin{aligned} p>\varPhi (kh-b)\Rightarrow \text {AF}=0 \end{aligned}$$. Follows from (13).
- $\varPhi (-b)<p<\varPhi (kh-b)\Rightarrow \text {AF}=\frac{\varPhi (-b)-\varPhi \left\{ \varPhi ^{-1}(p)-kh\right\} }{p},$ which decreases monotonically with *p*. Proof: Define $q=\varPhi ^{-1}(p)$. We then have that
  14 $$\begin{aligned} \text {AF}=\frac{\varPhi (-b)}{\varPhi (q)}-\frac{\varPhi (q-kh)}{\varPhi (q)}. \end{aligned}$$
  For the first term, we have that
  $$\begin{aligned} \frac{\partial }{\partial q}\left\{ \frac{\varPhi (-b)}{\varPhi (q)}\right\} <0. \end{aligned}$$
  For the second term, we have that
  $$\begin{aligned} \frac{\partial }{\partial q}\left\{ -\frac{\varPhi (q-kh)}{\varPhi (q)}\right\} =\frac{\phi (q)\varPhi (q-kh)-\varPhi (q)\phi (q-kh)}{\varPhi ^2(q)}. \end{aligned}$$
  This expression is negative if and only if
  $$\begin{aligned}&\phi (q)\varPhi (q-kh)-\varPhi (q)\phi (q-kh)<0\Leftrightarrow \\&\phantom {11}-\frac{\phi (q)}{\varPhi (q)}>-\frac{\phi (q-kh)}{\varPhi (q-kh)}>0, \end{aligned}$$
  Which is true, since $-\frac{\phi (q)}{\varPhi (q)}=E(X|X<q)>E(X|X<q-kh)=-\frac{\phi (q-kh)}{\varPhi (q-kh)}$, where *X* has a standard normal distribution. Thus, $\frac{\partial \text {AF}}{\partial q}<0\Rightarrow \frac{\partial \text {AF}}{\partial p}<0$, which concludes the proof.
- $p<\varPhi (-b)\Rightarrow \text {AF}=1-\frac{\varPhi \left\{ \varPhi ^{-1}(p)-kh\right\} }{p}$, which decreases monotonically with *p*, and similar proof as above.
- $$\lim _{p\rightarrow 0}\text {AF}=1$$. Proof:
  $$\begin{aligned} \lim _{p\rightarrow 0}\text {AF}= & {} \lim _{q\rightarrow -\infty }\text {AF}\\= & {} \lim _{q\rightarrow -\infty }1-\frac{\varPhi \left( q-kh\right) }{\varPhi (q)}\\= & {} \lim _{q\rightarrow -\infty }1-\frac{\partial \left\{ \varPhi \left( q-kh\right) /\partial q\right\} }{\partial \varPhi (q)/\partial q}\\= & {} \lim _{q\rightarrow -\infty }1-\frac{\phi (q-kh)}{\phi (q)}\\= & {} \lim _{q\rightarrow -\infty }1-\frac{(2\pi )^{-1/2}\text {exp}\left\{ -(q-kh)^2/2\right\} }{(2\pi )^{-1/2}\text {exp}\left\{ -q^2/2\right\} }\\= & {} \lim _{q\rightarrow -\infty }1-\text {exp}\left[ \{2qkh-(kh)^2\}/2\right] \\= & {} 1, \end{aligned}$$
  where the third equality follows from L’Hospital’s rule.
